# Use Patterns and Challenges of the Social Media Platform X Among Physiotherapists in Saudi Arabia: Cross-Sectional Study

**DOI:** 10.2196/84471

**Published:** 2026-01-23

**Authors:** Maryam Alasfour, Reem Alqahtani, Mohammed Amri, Sarah Alsultan, Salhah Hobani, Kholoud Almufaireej, Mohammad Alsinan

**Affiliations:** 1 Department of Medical Rehabilitation, Physical Therapy Department, Riyadh First Health Cluster, King Salman Hospital, 7790 Imam Abdulaziz bin Mohammed bin Saud, Riyadh, 7790, Saudi Arabia, 966 0540322703; 2 Department of Medical Rehabilitation, Physical Therapy Department, Riyadh First Health Cluster, Al-Quwayiyah Hospital, Al-Quwayiyah, Saudi Arabia; 3 Physiotherapy Department, Jazan Health Cluster, Sabya General Hospital, Jazan, Saudi Arabia; 4 Physical Therapy Department, Security Forces Hospital, Riyadh, Saudi Arabia; 5 Home Healthcare Administration, Saudi Ministry of Health, Riyadh, Saudi Arabia

**Keywords:** social media, professional development, physical therapists, knowledge management, health education, cross-sectional studies, evidence-based practice, Saudi Arabia, internet communication tools, social networking

## Abstract

**Background:**

Social media platforms have become salient channels for health care professionals’ continuous education and professional development. Among them, X (formerly known as Twitter) is used by physiotherapists for engaging in evidence-based discussions and accessing emerging research. In Saudi Arabia, a country with a high social media penetration rate, the platform offers unique opportunities and challenges for physiotherapy-related knowledge acquisition and networking.

**Objective:**

This study aimed to determine how physiotherapists in Saudi Arabia engage in physiotherapy-related debates on X, explore their use patterns, and identify associated challenges and perceived professional benefits.

**Methods:**

We conducted a cross-sectional online survey among licensed physiotherapists registered with the Saudi Commission for Health Specialties. The questionnaire covered demographic data, social media use, interaction patterns, perceived challenges, and motivations for use. Descriptive statistics and chi-square tests were used to examine demographic data, use patterns, challenges and concerns, perceived professional benefits, and the association between demographic characteristics and use patterns. Statistical significance was set at *P*<.05.

**Results:**

Of 193 responses, 188 (97.4%) were valid and included in the data analysis. Among the respondents, 76.1% (143/188) reported having an active account on X. Most respondents were female (109/188, 58.0%) and aged 31 to 40 years (79/188, 42.0%). The time spent on the platform varied, with 32.9% (47/143) spending 4 to 6 hours a week and 27.3% (39/143) spending less than an hour per week. Respondents’ interaction extent was moderate, with 35.7% (51/143) reporting occasional interaction. The respondents mainly interacted with knowledge-sharing posts (102/143, 71.3%), followed by training- or workshop-related posts (94/143, 65.7%). The respondents reported difficulty in finding reliable information (75/143, 52.4%), time constraints (58/143, 40.6%), communication barriers (69/143, 48.3%), and conflicts of interest (74/143, 51.7%) as challenges concerning engaging in physiotherapy-related debates on X. Despite these concerns, many respondents acknowledged the platform’s value as 60.1% (86/143) agreed that it helped them stay updated with emerging research, 68.5% (98/143) believed that it fostered knowledge sharing, and 67.8% (97/143) believed that it enhanced critical thinking among the community.

**Conclusions:**

Physiotherapists in Saudi Arabia demonstrated active engagement with physiotherapy-related content on X for professional development. While the platform offers valuable opportunities for learning and collaboration, notable barriers such as information credibility and time limitations must be addressed. Enhancing digital literacy and establishing clear guidelines for professional social media use may help maximize the platform’s potential as a tool for continuous development in physiotherapy practice.

## Introduction

Social media has transformed the way in which health care professionals engage in continuous learning, networking, and professional development [1]. Among the numerous social media platforms, X (formerly known as Twitter) serves as a prominent platform for discussions concerning evidence-based practice, clinical decision-making, and new research findings among physiotherapists. As health care disciplines become more digitally integrated, X and analogous platforms facilitate real-time knowledge exchange and professional dialogue [2,3]. Within the scope of digital professionalism, health care professionals are encouraged to engage in ethical, effective, and respectful online communication. When such professionalism is present, digital platforms such as X can promote professional participation, collaboration, and knowledge sharing. Online learning frameworks, particularly those presented by Anderson [4] and Garrison et al [5], underscore the significance of social presence, cognitive presence, and teaching presence in establishing a successful online learning environment. Therefore, platforms such as X can function as venues for professional learning via interaction and discussion. Furthermore, health communication theories such as the social cognitive theory [6] emphasize how individuals’ behaviors are influenced by observing others, particularly within online communities. Accordingly, health care professionals may adopt best practices and engage in evidence-based discussions through X.

While X provides opportunities for information dissemination and online learning, challenges such as content reliability, engagement barriers, and ethical concerns remain understudied, particularly in the context of physiotherapy practice in Saudi Arabia [1,7,8]. Saudi Arabia has one of the highest social media penetration rates worldwide [9-11], making it a fertile ground for exploring how digital platforms influence professional development among physiotherapists. Previous studies have examined the application of social media in health care [1,7,8]. However, research on the engagement patterns, challenges, and perceived benefits of X within the physiotherapy community remains limited [1,12,13]. Addressing this gap can provide insights into how physiotherapists use X for knowledge acquisition, professional networking, and overcoming practice-related challenges. Therefore, this study aimed to (1) assess the professional use of X among physiotherapists in Saudi Arabia (prevalence of active accounts, time spent on use, interaction level, and primary purpose of engagement) and (2) identify the challenges and motivations concerning physiotherapy-focused debates on X.

## Methods

### Design and Setting

This cross-sectional study was conducted in accordance with the Strengthening the Reporting of Observational Studies in Epidemiology (STROBE), Checklist for Reporting of Survey Studies (CROSS), and Checklist for Reporting Results of Internet E-Surveys (CHERRIES) guidelines to enhance methodological rigor, survey transparency, and reporting completeness. Data were collected via an online survey conducted among licensed physiotherapists practicing across various health care settings. Furthermore, we adhered to established research guidelines to ensure validity and reliability.

### Participants and Recruitment

This study targeted licensed physiotherapists who were registered with the Saudi Commission for Health Specialties (SCFHS) and were actively working in Saudi Arabia. Physiotherapy students, interns, and professionals without an active SCFHS registration were excluded. Using convenience sampling, we distributed participation invitations within physiotherapy-focused professional groups on social media platforms such as WhatsApp, Telegram, and X. The invitation stated the following: “Licensed physiotherapists in Saudi Arabia are invited to participate in a voluntary and anonymous survey examining physiotherapists’ use of X for professional development.” This wording was consistent across all channels used for dissemination. Additionally, the invitation described the study’s purpose and eligibility criteria.

### Survey Instrument

A structured questionnaire was developed based on a comprehensive review of relevant literature and prior studies investigating social media engagement in health care [8,12-18]. This review provided the initial pool of survey items covering use patterns, interaction behaviors, and challenges and motivations related to engagement on X. Consequently, the questionnaire comprised 4 main sections.

Through 6 items, the first section collected demographic information (age, sex, years of experience, highest level of education, and workplace setting [government hospital, private clinic, private hospital, or academic institution]) and whether the respondent had an active account on X. The second section comprised 3 items and collected data on social media use and interaction: frequency of use, time spent on X, preferred content type (eg, debates, knowledge sharing, mentorship, and training), and pattern of engagement. The third section consisted of 5 items and collected data on challenges and concerns regarding engaging in physiotherapy-related debates on X, such as time constraints, information credibility, communication barriers, and conflicts of interest. The final section comprised 5 items and explored the motivations for and perceived benefits of following physiotherapy-related debates on X.

The questionnaire included a combination of multiple-choice questions and Likert-scale items. We conducted a pilot test among 10 to 15 licensed physiotherapists to assess clarity, comprehension, flow, and item relevance. On the basis of their feedback on phrasing, ease of completion, and the appropriateness of the response options, we refined several items for clarity, removed ambiguous statements, and reordered questions for readability and coherence. The final version demonstrated satisfactory internal consistency (Cronbach α=0.87), ensuring reliability and content validity before full deployment.

### Data Collection

The survey was conducted on SurveyMonkey (SurveyMonkey Inc) from January 5, 2024, to February 10, 2024. The participants were required to provide informed consent before starting the survey. The survey spanned 5 pages, which included the consent page, demographic items, use questions, and challenge- and motivation-related items. The estimated completion time was 10 to 15 minutes. To ensure data quality and prevent duplicate responses, we activated SurveyMonkey’s built-in “Prevent multiple submissions” feature (cookie based) and enabled IP address tracking and time stamp checks. The IP addresses were automatically collected by the platform for quality control but were not analyzed, linked to individual responses, or included in the exported dataset.

### Data Analysis

Descriptive statistics (frequencies and percentages) were used to summarize participant characteristics and patterns of professional use of X. Next, we examined the association between demographic characteristics and use patterns using chi-square tests. Because the variables in this study were primarily categorical, no *t* tests or ANOVA were conducted. Missing data were assessed to determine the underlying mechanism, and patterns suggested that data were missing completely at random as there was no systematic association between missingness and participant characteristics. Therefore, pairwise (available-case) deletion was used to handle missing values rather than imputation. Subsequently, inferential analyses were performed to explore associations between physiotherapists’ demographic characteristics and their professional use of X. Statistical significance was set at *P*<.05, and all analyses were conducted using SPSS Statistics (version 28; IBM Corp).

### Ethical Considerations

This study complied with the principles outlined in the Declaration of Helsinki. Ethics approval was obtained from the King Saud Medical City Institutional Review Board (H-01-R-053). Participation was entirely voluntary, and informed consent was obtained electronically before the start of the survey. To ensure privacy and confidentiality, responses were anonymized before analysis. SurveyMonkey automatically collected IP addresses for quality control; however, these addresses were not analyzed, linked to responses, or included in the exported dataset. Participants did not receive any financial or nonfinancial compensation for taking part.

## Results

### Characteristics of the Participants

A total of 193 individuals accessed the survey. However, of these 193 individuals, 1 (0.5%) refused to participate, and 4 (2.1%) were not SCFHS-registered physiotherapists practicing in Saudi Arabia. After excluding these individuals, 97.4% (188/193) of the responses were used for data analysis (response rate consistent with the CROSS guidelines). Most participants were female (109/188, 58.0%) and in the 31- to 40-year age group (79/188, 42.0%). Regarding work experience, 38.8% (73/188) of the participants reported 11 or more years of experience, 36.7% (69/188) reported 0 to 5 years, and 24.5% (46/188) reported 6 to 10 years. Most participants held a bachelor’s degree (132/188, 70.2%), followed by a master’s degree (43/188, 22.9%) and a PhD (13/188, 6.9%). Most of the participants (113/188, 60.1%) worked in governmental hospitals, whereas 16.5% (31/188) worked in private clinics, 13.3% (25/188) worked in private hospitals, and 10.1% (19/188) worked in academic institutions. Furthermore, most participants (143/188, 76.1%) reported having an active account on X (Table 1).

**Table 1.** Demographic and professional characteristics of licensed physiotherapists participating in a cross-sectional survey conducted in Saudi Arabia between January 2024 and February 2024 (n=188).

Characteristic and categories	Participants, n (%)
Age (years)	
20‐30	66 (35.1)
31‐40	79 (42.0)
41‐50	31 (16.5)
51‐60	12 (6.4)
Sex	
Female	109 (58.0)
Male	79 (42.0)
Experience (years)	
0‐5	69 (36.7)
6‐10	46 (24.5)
≥11	73 (38.8)
Highest level of education	
Bachelor’s degree	132 (70.2)
Master’s degree	43 (22.9)
PhD	13 (6.9)
Workplace setting	
Academic institution	19 (10.1)
Governmental hospital	113 (60.1)
Private clinic	31 (16.5)
Private hospital	25 (13.3)
Active account on X	
No	45 (23.9)
Yes	143 (76.1)

### Patterns of Professional Use of X Among Physiotherapists in Saudi Arabia

Regarding the time spent per week following or viewing professional physiotherapy-related posts, 32.9% (47/143) of the participants spent 4 to 6 hours, 27.3% (39/143) spent less than an hour, and 25.9% (37/143) spent 1 to 3 hours. Regarding the extent of interaction with physiotherapy-related debates, 35.7% (51/143), 27.3% (39/143), and 18.2% (26/143) of the participants interacted occasionally, rarely, and frequently, respectively. Furthermore, the participants predominantly interacted with knowledge sharing–related (102/143, 72.3%), training- or workshop-related (94/143, 66.7%), or debate-related (37/143, 26.2%) posts (Table 2).

**Table 2.** Patterns of professional use of X among licensed physiotherapists in Saudi Arabia based on a cross-sectional survey conducted between January 2024 and February 2024 (n=143).

Item and categories	Participants, n (%)
“How much time per week do you spend following or viewing professional physiotherapy-related posts on X?”	
Less than an hour	39 (27.3)
1‐3 hours	37 (25.9)
4‐6 hours	47 (32.9)
7‐10 hours	11 (7.7)
More than 10 hours	9 (6.3)
“Please indicate the extent to which you interact with physiotherapy-related debates on X. (Interaction refers to commenting, reposting, liking, quoting, bookmarking, or sharing)”	
Very frequently	3 (2.1)
Frequently	26 (18.2)
Occasionally	51 (35.7)
Rarely	39 (27.3)
Not at all	24 (16.8)
“What types of professional posts do you interact with? (Please select all that apply). (Interaction refers to commenting, reposting, liking, quoting, bookmarking, or sharing)”	
Debates	37 (25.9)
Knowledge sharing	102 (71.3)
Collaborative projects	33 (23.1)
Teamwork or collaboration	25 (17.5)
Training or workshops	94 (65.7)
Mentorship or mentoring	11 (7.7)
I do not interact (only follow or view posts)	29 (20.3)

### Association Between Demographic Characteristics and Use Patterns

#### Demographic Characteristics and Time Spent on the Platform

The chi-square tests revealed that the time spent following or viewing professional physiotherapy-related posts was significantly associated with sex (*χ*^2^_4_=18.8; *P*=.001) and workplace setting (*χ*^2^_12_=24.2; *P*=.02). In contrast, the time spent was not significantly associated with the highest level of education (*P*=.13), as shown in Table 3.

**Table 3.** Associations between demographic characteristics and patterns of engagement with physiotherapy-related debates on X among licensed physiotherapists in Saudi Arabia (cross-sectional study, 2024).

Demographic characteristic and outcome variable	Chi-square (*df*)	*P* value
Sex		
Time spent	18.8 (4)	.001
Interaction extent	7.7 (4)	.10
Highest level of education		
Time spent	12.4 (8)	.13
Interaction extent	7.1 (8)	.52
Workplace setting		
Time spent	24.2 (12)	.02
Interaction extent	27.7 (12)	.006

#### Demographic Characteristics and Extent of Interaction

The extent of interaction (eg, liking, sharing, and commenting) was significantly associated with workplace setting (*χ*^2^_12_=27.7; *P*=.006). However, it was not significantly associated with sex (*χ*^2^_4_=7.7; *P*=.10) or highest level of education (*χ*^2^_8_=7.1; *P*=.52; Table 3).

### Challenges and Concerns Regarding Engaging in Physiotherapy-Related Debates on X

Among the respondents, 49.0% (70/143) agreed or strongly agreed that they had encountered challenges or negative experiences, with 31.5% (45/143) remaining neutral on this issue. Lack of time was also a significant concern, with 40.6% (58/143) agreeing or strongly agreeing that it affected their interaction with physiotherapy-related debates, although a third of the respondents (48/143, 33.6%) disagreed or strongly disagreed. Finding relevant and reliable information was another challenge, with 52.4% (75/143) of the respondents agreeing or strongly agreeing that they experienced this issue, whereas 36.4% (52/143) remained neutral. Communicating ideas effectively was problematic for 48.3% (69/143) of the respondents, whereas 51.7% (74/143) reported experiencing conflicts of interest related to their personal or professional involvement (Table 4).

**Table 4.** Perceived challenges and concerns regarding engaging in physiotherapy-related debates on X among licensed physiotherapists in Saudi Arabia based on a cross-sectional survey conducted in 2024 (n=143).

Item and categories	Participants, n (%)
“I have encountered challenges or negative experiences while interacting with or following physiotherapy-related debates on X.”	
Strongly disagree	7 (4.9)
Disagree	21 (14.7)
Neutral	45 (31.5)
Agree	59 (41.3)
Strongly agree	11 (7.7)
“Lack of time is a challenge that affects my interaction with physiotherapy-related debates on X.”	
Strongly disagree	18 (12.6)
Disagree	30 (21.0)
Neutral	37 (25.9)
Agree	44 (30.8)
Strongly agree	14 (9.8)
“I have experienced challenges or difficulties in finding relevant and reliable information while following physiotherapy-related debates on X.”	
Strongly disagree	2 (1.4)
Disagree	14 (9.8)
Neutral	52 (36.4)
Agree	55 (38.5)
Strongly agree	20 (14.0)
“I have experienced challenges or difficulties in communicating ideas effectively while participating in physiotherapy-related debates on X.”	
Strongly disagree	3 (2.1)
Disagree	26 (18.2)
Neutral	45 (31.5)
Agree	51 (35.7)
Strongly agree	18 (12.6)
“I have experienced personal or professional conflicts of interest while interacting with or following physiotherapy-related debates on X.”	
Strongly disagree	2 (1.4)
Disagree	19 (13.3)
Neutral	48 (33.6)
Agree	52 (36.4)
Strongly agree	22 (15.4)

### Motivations for and Perceived Benefits of Following Physiotherapy-Related Debates on X

As shown in Table 5, a significant proportion of physiotherapists (60%‐70%) saw value in engaging with debates on X as it allowed them to stay informed about new research; catered to their personal interests; and provided potential benefits for the wider physiotherapy community in terms of knowledge sharing, collaboration, and critical thinking. Among the respondents, 39.2% (56/143) agreed and 21.0% (30/143) strongly agreed that they engaged in physiotherapy-related debates because it offered them the opportunity to keep up with new and emerging research. Meanwhile, 39.2% (56/143) agreed and 23.8% (34/143) strongly agreed that they were motivated by their personal interest in the topics being discussed. Similarly, 42.0% (60/143) agreed and 26.6% (38/143) strongly agreed that the debates could facilitate knowledge sharing, collaboration, and networking among the physiotherapist community. Furthermore, 42.7% (61/143) agreed and 25.2% (36/143) strongly agreed that the debates could promote critical thinking and reflection on current practices. A total of 29.4% (42/143) to 33.6% (48/143) of the respondents remained neutral on these issues.

**Table 5.** Motivations for and perceived benefits of following physiotherapy-related debates on X among licensed physiotherapists in Saudi Arabia based on a cross-sectional survey conducted in 2024 (n=143).

Item and categories	Participants, n (%)
“I follow or interact with physiotherapy-related debates on X because it allows me to keep up with new and emerging research.”	
Strongly disagree	3 (2.1)
Disagree	12 (8.4)
Neutral	42 (29.4)
Agree	56 (39.2)
Strongly agree	30 (21.0)
“I follow or interact with physiotherapy-related debates on X because I have an interest in the topics being discussed.”	
Strongly disagree	2 (1.4)
Disagree	12 (8.4)
Neutral	39 (27.3)
Agree	56 (39.2)
Strongly agree	34 (23.8)
“Following or interacting with physiotherapy-related debates on X can facilitate knowledge- and experience-sharing among the physiotherapist community, thereby fostering collaboration and networking.”	
Strongly disagree	2 (1.4)
Disagree	5 (3.5)
Neutral	38 (26.6)
Agree	60 (42.0)
Strongly agree	38 (26.6)
“Following or interacting with physiotherapy-related debates on X can promote critical thinking and reflection on current practices among the physiotherapist community.”	
Strongly disagree	1 (0.7)
Disagree	8 (5.6)
Neutral	37 (25.9)
Agree	61 (42.7)
Strongly agree	36 (25.2)
“To what extent would you recommend physiotherapy students or colleagues to follow or interact with physiotherapy-related debates on X?”	
Strongly disagree	2 (1.4)
Disagree	9 (6.3)
Neutral	48 (33.6)
Agree	48 (33.6)
Strongly agree	36 (25.2)

## Discussion

### Principal Findings

The findings of this study align with those of previous research indicating that social media platforms, particularly X, serve as valuable tools for professional development and knowledge sharing among health care professionals [12,13]. Similar to prior studies, this study found that physiotherapists in Saudi Arabia actively use X for knowledge acquisition and training opportunities [17,18]. However, as this cross-sectional survey–based study collected self-reported data, the associations should be interpreted with caution, and causal inferences cannot be drawn about practice changes. The high engagement rate suggests that X is widely accepted as an informal learning tool and it may complement traditional educational methods in physiotherapy [16].

However, this study also highlighted significant challenges associated with the use of social media for professional development. Concerns about information credibility and time constraints were commonly reported, mirroring the findings of studies involving other health care professionals [14,15]. Moreover, previous studies have reported that the difficulty in distinguishing between evidence-based content and misinformation hinders effective engagement on health care–related social media channels [19]. Addressing these concerns through improved content curation and institutional guidelines may enhance the usability of X as a source of professional development. Furthermore, recent studies, particularly those in the health care field, have reported concerns about unprofessional behavior on social media. Vukušić Rukavina et al [20] emphasized the importance of defining and addressing unprofessional behaviors online to ensure that health care professionals maintain ethical standards in digital spaces.

Similar to our findings, Alasfour et al [1] reported that, while X positively affects physiotherapists’ professional development, a substantial portion of users remain passive consumers rather than active participants in discussions. In this study, demographic variables such as sex and workplace setting strongly influenced the time spent on the platform. This finding aligns with those of other research suggesting that personal motivation or digital literacy may play a larger role in determining platform use patterns, which reflects global trends in health care professionals’ social media engagement [7,21]. Future efforts should increase active participation through strategies such as structured online discussions, mentorship programs, and incentives for contribution. Social media presents not only benefits but also potential risks for health care professionals. Vukušić Rukavina et al [22] reviewed the dangers and benefits of social media use, stressing the potential for professional growth and networking while cautioning against the risk of diminished professionalism if used improperly. These findings underscore the necessity of establishing professional guidelines and enhancing digital literacy to maximize the advantages of online engagement while safeguarding against its potential pitfalls.

The recommendations in this study align with those of previous research advocating for clearer professional guidelines on social media use for physiotherapy professional growth [12,13]. Establishing best practices and offering professional training on effective digital communication can further enhance the platform’s role in facilitating continuous learning and professional networking. Overall, the findings reinforce the importance of social media as an emerging tool for physiotherapy education while emphasizing the need for addressing engagement barriers to maximize its potential.

### Limitations

This study has several limitations. First, the use of convenience sampling may have introduced selection bias and limited generalizability. Because recruitment was conducted through physiotherapy-focused social media groups, the sample likely overrepresented physiotherapists already active on social media platforms. Consequently, the findings may not fully reflect the behaviors and perspectives of all SCFHS-registered physiotherapists, particularly those who are less digitally engaged or not present in such online communities. Second, the reliance on self-reported data may have led to recall or social desirability bias. Third, while inferential tests were conducted, the study’s design was descriptive. Therefore, these analyses should be interpreted with caution. Future studies should use probability sampling or mixed methods approaches.

### Conclusions

There is a high prevalence of active X accounts among SCFHS-registered licensed physiotherapists. With 1 to 6 hours a week spent on the platform, they exhibited high levels of engagement with physiotherapy-related debates on X. While the platform offers substantial benefits, it also presents challenges that must be addressed to maximize its potential as a professional tool. By understanding and addressing these challenges, X can become an effective resource for physiotherapists, contributing to their ongoing development and improving patient care outcomes.

## Abbreviations

CHERRIES: Checklist for Reporting Results of Internet E-Surveys
CROSS: Checklist for Reporting of Survey Studies
SCFHS: Saudi Commission for Health Specialties
STROBE: Strengthening the Reporting of Observational Studies in Epidemiology
